# Production of Recombinant Replication-defective Lentiviruses Bearing the SARS-CoV or SARS-CoV-2 Attachment Spike Glycoprotein and Their Application in Receptor Tropism and Neutralisation Assays

**DOI:** 10.21769/BioProtoc.4249

**Published:** 2021-11-05

**Authors:** Nazia Thakur, Giulia Gallo, Ahmed M. E. Elreafey, Dalan Bailey

**Affiliations:** 1 Viral Glycoproteins Group, The Pirbright Institute, Pirbright, Woking, UK; 2 The Jenner Institute, Nuffield Department of Medicine, University of Oxford, Oxford, UK

**Keywords:** SARS-CoV-2, SARS-CoV, Pseudotyped virus, Tropism, Neutralisation

## Abstract

For enveloped viruses, such as SARS-CoV-2, transmission relies on the binding of viral glycoproteins to cellular receptors. Conventionally, this process is recapitulated in the lab by infection of cells with isolated live virus. However, such studies can be restricted due to the availability of high quantities of replication-competent virus, biosafety precautions and associated trained staff. Here, we present a protocol based on pseudotyping to produce recombinant replication-defective lentiviruses bearing the SARS-CoV or SARS-CoV-2 attachment Spike glycoprotein, allowing the investigation of viral entry in a lower-containment facility. Pseudoparticles are produced by cells transiently transfected with plasmids encoding retroviral RNA packaging signals and *Gag-Pol* proteins, for the reconstitution of lentiviral particles, and a plasmid coding for the viral attachment protein of interest. This approach allows the investigation of different aspects of viral entry, such as the identification of receptor tropism, the prediction of virus host range, and zoonotic transmission potential, as well as the characterisation of antibodies (sera or monoclonal antibodies) and pharmacological inhibitors that can block entry.

Graphic abstract:

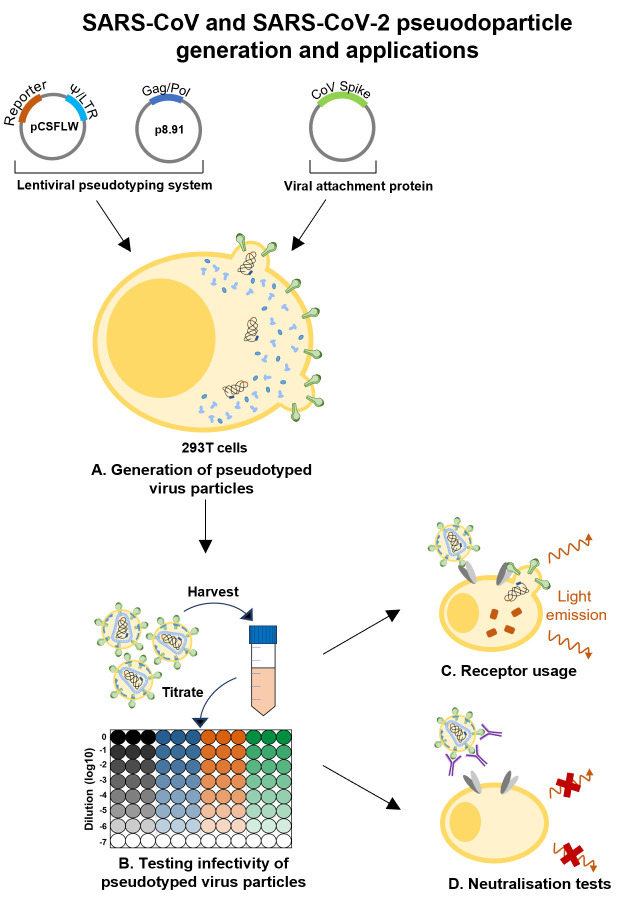

SARS-CoV and SARS-CoV-2 pseudoparticle generation and applications.

## Background

Pseudoparticles are replication-defective viral particles obtained through expression of viral envelope glycoproteins on the surface of a recombinant virus, which provides the core components of the particle. Vesicular stomatitis virus (VSV), a rhabdovirus, and two lentiviruses – human immunodeficiency virus-1 (HIV-1) and murine leukaemia virus (MuLV) – are commonly used as viral vectors for pseudotyping (Takada *et al.*, 1997; Wool-Lewis and Bates, 1998; Sharkey *et al.*, 2001; Negrete *et al.*, 2005; Grehan *et al.*, 2015; Thakur *et al.*, 2021). In our recent study, we successfully used a lentiviral-based system to study the interaction of severe acute respiratory syndrome coronavirus (SARS-CoV) and SARS-CoV-2 Spike (S) protein with its cellular receptor, angiotensin converting enzyme 2 (ACE2) (Conceicao *et al.*, 2020).

SARS-CoV-2, the etiological agent of the ongoing COVID-19 pandemic, is a highly pathogenic betacoronavirus that requires handling at BSL-3 facilities, which are not always available in research laboratories. To allow work with SARS-CoV and SARS-CoV-2 at lower containment, the generation of viral particles pseudotyped with the Spike protein represents a safe and appealing surrogate. This technique allows (i) dissection of viral entry pathways, (ii) investigation of host cell susceptibility and tropism of the angiotensin converting enzyme 2 (ACE2) receptor, (iii) examination of inter-species transmission, (iv) assessment of the neutralising antibody responses in immunogenicity and sero-epidemiological studies, and (v) efficacy assessment of small-molecule inhibitors that block viral entry. Notably, this technique has been applied to viral glycoproteins from a wide variety of viruses such as influenza hemagglutinin (Bertram *et al.*, 2010), Nipah virus fusion and attachment proteins (Thakur *et al.*, 2021), Ebola virus glycoprotein (Simmons *et al.*, 2003), Chikungunya virus E1 (Salvador *et al.*, 2009), hepatitis C virus E2 proteins (Hsu *et al.*, 2003), and VSV glycoprotein (DePolo *et al.*, 2000).

To generate lentiviral-based pseudoparticles of HIV-1, cells are co-transfected with the following plasmids: (i) HIV-1 packaging plasmid encoding for the core genes *Gag* and *Pol*, (ii) the transfer plasmid that encodes a firefly luciferase reporter gene flanked by HIV-1 regulatory LTR regions and the packaging signal, and (iii) a third plasmid encoding for the heterologous viral glycoprotein. Pseudoparticles possessing the viral glycoprotein of interest on their surface are assembled at the cellular membrane, from which they bud (Zufferey *et al.*, 1997). Upon infection, the luciferase gene encoded by the lentivirus genome is expressed, allowing accurate quantification of viral entry.

## Materials and Reagents

1. 50 ml Falcon tubes (VWR International, catalog number: 734-0448)
2. Clear bottom 6-well tissue-culture treated plate (Scientific Laboratory Supplies, Falcon^TM^, catalog number: 353046)
3. Pipette tips (STARLAB, catalog numbers: S1110-3700 [10/20 µl XL Graduated TipOne^®^]; S1111-1206-C [200 µl Yellow Bevelled TipOne^®^ Tip]; S1112-17200 [1,250 µl XL Graduated TipOne^®^])
4. Serological pipettes (Corning, catalog numbers: 4101 [10 ml Stripette^TM^]; 4051 [5 ml Stripette^TM^]; 4251 [25 ml Stripette^TM^])
5. Opti-MEM^TM^ (Thermo Scientific, Gibco^TM^, catalog number: 11058021, storage conditions: 4°C, shelf life: 12 months)
6. Disposable weighing boats 85 × 85 × 24 mm, PS, medium, white, anti-static (VWR, catalog number: 10770-448, storage conditions: room temperature)
7. 7 ml polycarbonate polypropylene screw cap bijous (container for storage of small-volume samples) (STARLAB, catalog number: E1412-0710)
8. 96-well Delta-treated (hydrophilic surface that promotes cell attachment and growth) White flat-bottom plate (Fisher Scientific, Nunc, MicroWell, catalog number: 10182831)
9. Tissue culture flasks (Greiner Bio-One, catalog numbers: 660160 [175 cm^2^], 658170 [75cm^2^])
10. 1.5 ml Microcentrifuge sterile Eppendorf tubes (STARLAB, TubeOne^®^, catalog number: S1615-5510)
11. Millex-GP syringe filter unit, 0.22 µm filter, polythersulfone, 33 mm, gamma sterilised (Merck, Millipore, catalog number: SLGP033RS, storage conditions: room temperature)
12. Human Embryonic Kidney 293T, HEK293T cells (ATCC^®^, catalog number: CRL-3216^TM^, storage conditions: liquid nitrogen vapour phase)
13. Baby Hamster Kidney-21, BHK-21 cells (ATCC^®^, catalog number: CCL-10^TM^, storage conditions: liquid nitrogen vapour phase)
14. Plasmid DNA: ACE2 receptors (pDISPLAY expression vector, codon-optimised, N-terminal signal peptide [the murine Ig κ-chain leader sequence], C-terminal HA-tag), SARS-CoV Spike, SARS-CoV-2 Spike (pcDNA3.1(+), codon-optimised, C-terminal FLAG-tag) (BioBasic, Canada [Conceicao *et al.*, 2020]), p8.91, CSFLW, VSV-G (pcDNA3.1(+) expression vector) (available upon request), pcDNA3.1(+) (Thermo Scientific, Invitrogen, catalog number: V79020) and pDISPLAY^TM^ (Thermo Scientifc, Invitrogen^TM^, catalog number: V66020)
15. Sera or antibodies for neutralisation assays, with relevant biological risk assessment and ethical approvals in place
16. Dulbecco’s modified Eagle’s medium, DMEM (Merck, Sigma-Aldrich, catalog numbers: D5796 [with phenol red]; D1145 [phenol red free], storage conditions: 4°C, 12 months)
17. Foetal bovine serum, FBS (Life Science Production, catalog number: S-001A-BR, -20°C)
18. Penicilin-Streptomycin, 10,000 U/ml (Thermo Scientific, Gibco^TM^, catalog number: 15240122, storage conditions: -20°C, shelf life: 12 months)
19. Sodium pyruvate, 100 mM (Thermo Scientific, Gibco^TM^, catalog number: 11360070, storage conditions: 4°C, shelf life: 12 months)
20. EDTA (0.5 M), pH 8.0, RNase-free (Thermo Scientific, Ambion^®^, catalog number: AM9269G)
21. 1× Trypsin-EDTA, 0.25%, phenol red (Thermo Scientific, Gibco^TM^, catalog number: 2520072, storage conditions: -20°C long-term, 4°C while in use, shelf life: 24 months)
22. *Trans*IT-X2^®^ Dynamic Delivery System (Mirus, catalog number: MIR 6000, storage conditions: -20°C, shelf life: 12 months)
23. Polyethyleneimine, PEI (Merck, Sigma-Aldrich, catalog number: 408727, storage conditions: 4°C)
24. Nuclease-free, autoclaved, 0.2 µm filtered DEPC-treated water (Ambion, catalog number: AM9906, storage conditions: room temperature)
25. Hydrochloric acid 36.5-38.0%, Bioreagent, for molecular biology (Sigma-Aldrich, catalog number: H1758-100 ml, storage conditions: room temperature)
26. Bright-Glo^TM^ Luciferase Assay System (Promega, catalog number: E2650, storage conditions: -20°C)
27. 55 ml StarTub PVC reagent reservoirs (STARLAB, sterile individually wrapped, catalog number: E2310-1010)
28. DMEM-10% (see Recipes)
29. Working solution of 1 mg/ml PEI (see Recipes)

## Equipment

1. Microbiological safety cabinet, BSL-2 (CAS, Biomat 2 – class 2 complies with BS EN 12469:2000)
2. CO_2_ incubator (PHC Europe B.V., PHCbi, catalog number: MCO-170AICD-PE)
-86°C ultra-low temperature freezer (PHCbi, Panasonic, vip plus, model: MDF-DU900V)
-20°C Medical freezer with 14 storage drawers (Liebherr, Profiline, model: G5216)
5. 4°C refrigerator (VDW CoolSystems, Labcold, Sparkfree, model: RLV0217)
6. Sub aqua 5 plus water bath (Fisher Scientific, Grant, model: 13251183)
7. Automated pipettor for serological pipettes (Fisher Scientific, Thermo Scientific^TM^, S1 Pipet Fillers, catalog number: 10072332)
8. Single-channel pipettes (Gilson, Pipetman L, catalog numbers: FA1001M [P2L 0.2-2 µl], FA1003M [P20L 2-20 µl]; FA1005M [P200L 20-200 µl]; FA1006M [P1000L 100-1,000 µl])
9. Multi-channel pipettes (Thermo Scientific^TM^, Finnpipette^TM^ F2 multichannel pipette, catalog numbers: 4662010 [8-well 5-50 µl]; 4662070 [12-well 30-300 µl])
10. Inverted microscope for cell culture (Leica microsystems, model: DMi1-S 40/0.45)
11. Haemocytometer (Fisher Scientific, Hirschmann^TM^ Bright Lined Counting Chambers, catalog number: 105289616)
12. Centrifuge machine (Kendo laboratory product, Sorvall Legend RT, EASYset, model: 75004373)
13. Benchtop autoclave (Fisher Scientific, Astell scientific, catalog number: 12755375)
14. GloMax^®^ Discover Microplate Reader (Promega, catalog number: GM3000)

## Software

1. Microsoft Excel (Microsoft 365 for Windows, www.microsoft.com)
2. GraphPad Prism (Version 8.4.2, GraphPad Software for Windows, San Diego, California USA, www.graphpad.com)
3. GloMax^®^ Discover System Software (Version 3.2.3, Promega, Southampton, United Kingdom www.promega.co.uk)

## Procedure

- Generation of SARS-CoV-2 and SARS-CoV pseudotyped virus particles
  1. Maintain HEK293T cells for pseudoparticle production in 25 ml of DMEM-10% (see Recipes) in a 75 cm^2^ tissue culture flask.
  2. Seed HEK293T cells at a concentration of 7.5 × 10^5 ^cells per well in a 6-well plate in 3 ml of DMEM-10%, for the total number of wells required.
  3. Agitate cells in the plate using a rapid up-down, left-right movement. This will ensure cells are evenly distributed and do not clump. Incubate at 37°C, 5% CO_2_ overnight.
  4. The next day, set up transfection mixes in the afternoon. The seeded HEK293T cells should be between 60-80% confluent for optimal transfection efficiency. Set up transfections for the SARS-CoV-2 S or SARS-CoV S plasmid, alongside an empty vector negative control (no glycoprotein, no GP) and a positive control. For instance, if the SARS-CoV-2 S and SARS-CoV S plasmids are in a pcDNA3.1 backbone, use an empty pcDNA3.1 plasmid as your no GP control. Generally, a VSV-G plasmid is used as a positive control, as it is trans-encapsidated into the HIV-1 particle efficiently (*i.e*., it pseudotypes well).
  5. In a sterile 1.5 ml Eppendorf tube, add 100 µl of Opti-MEM along with 0.6 µg of p8.91 plasmid (encoding for HIV-1 gag-pol), 0.6 µg of CSFLW plasmid (lentivirus backbone expressing Firefly luciferase), and 0.5 µg of glycoprotein (SARS-CoV-2 S, SARS-CoV S or VSV-G) or empty vector (pcDNA3.1) per well. Incubate for 5 min at room temperature.
  6. In a separate 1.5 ml Eppendorf tube, add 100 µl of Opti-MEM plus and 10 µl of PEI (1 µg/ml) per transfection and incubate for 5 min at room temperature.
  7. For each 100 µl transfection mix of DNA in Opti-MEM, add 100 µl of PEI in Opti-MEM and mix vigorously with a pipette ten times. Incubate at room temperature for 20 min.
  8. Add 200 µl of the volume of the transfection mix in a dropwise manner to each well of the 6-well plate and incubate overnight at 37°C, 5% CO_2_.
  9. The next morning, use a serological pipette to gently remove the media from wells containing the transfection mix by tilting the dish towards you and aspirating from the edge of the well, being careful not to disturb the monolayer. Replace with 3 ml of DMEM-10%. Incubate overnight at 37°C, 5% CO_2_ for 24 h.
  10. Harvest cell supernatants containing pseudotyped virus particles and transfer to a 50 ml Falcon, pooling similarly transfected wells, and store at 4°C. Replace the media with 3 ml of DMEM-10% per well, and incubate at 37°C, 5% CO_2_ for 24 h.
  11. Harvest the cell supernatants containing pseudotyped virus particles and pool with pseudoparticles harvested the day before. Centrifuge at 2,500 *× g* for 10 min at 4°C to remove cellular debris.
  12. Aliquot 4-5 ml of pseudoparticles into bijous and freeze at -80°C until further use.
*
NB: Larger volume of pseudoparticles can also be prepared in 10 cm^2^ culture dishes. The necessary cell seeding density, DNA concentrations and volumes required for this setup can be found in* ***Table 1***, *with the corresponding values for the 6-well plate format noted alongside. Steps A9-A12 remain the same regardless of the dish size used, changing only the volume of media required.*
  **Table 1.** Quick-guide to generating lentiviral-based pseudotyped viruses

		6-well plate format	10 cm^2^ dish format
**Cell seeding density**	HEK293T cells	7.5 × 10^5^ per well in 3 ml total volume DMEM-10%	2 × 10^6^ per culture dish in 10 ml total volume DMEM-10%
**DNA mix**	*Viral glycoprotein:* SARS-CoV-2/SARS-CoV Spike **OR** pcDNA3.1 empty vector (NE, negative control) **OR** VSV-G (positive control)	0.9 µg	1 µg
	p8.91 (HIV-1 gag/pol)	0.6 µg	1 µg
	pCSFLW (HIV-1 LTR, Firefly luciferase gene)	0.6 µg	1.5 µg
	Opti-MEM	100 µl	200 µl
**Transfection reagent mix**	PEI (1 µg/ml)	10 µl	20 µl
	Opti-MEM	100 µl	200 µl
**Total volume per well**	Opti-DNA + Opti-PEI mix	200 µl	400 µl
**Media**	Volume of DMEM-10% to replace after transfection or harvest	3 ml	10 ml
- Testing SARS-CoV-2 and SARS-CoV pseudoparticle infectivity
  1. Seed HEK293T cells at a density of 7.5 × 10^5^ per well in a 6-well plate in a total of 3 ml of DMEM-10%. Incubate overnight at 37°C, 5% CO_2_.
  2. Ensure plated cells are at 60-80% confluency to ensure optimal transfection efficiency. Set up transfection mixes to test pre-generated SARS-CoV-2 pseudoparticles. In a sterile 1.5 ml Eppendorf tube, add 200 µl of Opti-MEM along with 500 ng of human ACE2 plasmid per well to be transfected. Bring the Tran*IT*-X2 transfection reagent to room temperature before use, add 2 µl (for every 1 µg of DNA) directly to the tube, and gently flick the tube to mix. Incubate at room temperature for 20 min.
  3. Add 200 µl of the transfection mix dropwise to each well of the pre-plated cells and incubate overnight at 37°C, 5% CO_2_.
  4. Remove the media containing the transfection mix from the wells by tilting the dish towards you and aspirating from the edge of the well using a serological pipette, being careful not to disturb the monolayer. Add 1 ml of DMEM-10% per well and harvest the transfected cells. HEK293T cells have low adherence and come off the plate easily. As such, using the force of the pipetted liquid is sufficient to harvest cells, although care should be taken to ensure a single cell suspension is achieved without clumps. Trypsin should be avoided as this will unnecessarily cleave off the receptors, hampering future experimentation. Transfer to a 50 ml Falcon and dilute cells to 2 × 10^5^/ml with DMEM-10%.
  5. Seed 100 µl of diluted cells (2 × 10^4^ per well) into a flat, white-bottomed 96-well plate and incubate overnight at 37°C, 5% CO_2_.
  6. The next day, thaw an aliquot of SARS-CoV-2 and/or SARS-CoV pseudoparticles, along with the negative (pcDNA3.1, no GP) and positive (VSV-G) controls. Titrate the pseudoparticles in a clear-bottomed 96-well plate, starting with undiluted virus in the top row, and titrating 10-fold in DMEM-10%, for a final volume of 100 µl.
  7. Gently remove the media from the white-plate seeded with human ACE2-transfected cells and add 100 µl titrated pseudoparticles. Incubate for 48 h at 37°C, 5% CO_2_.
  8. Remove the media from the wells by tilting the dish towards you and aspirating from the edge of the well using a multi-channel pipette and add 50 µl Bright-Glo^TM^ diluted 1:1 with serum free, phenol red free DMEM. Incubate the plate in the dark for 5 min and then measure the luciferase signal on a GloMax Multi+ Detection System under the luminescence protocol with 0.5 s integration.
  9. Export the CSV file generated on a USB flash drive for analysis using Microsoft Excel and plot data on GraphPad Prism.
- ACE2 receptor usage screen using SARS-CoV-2 and SARS-CoV pseudotyped virus particles (Conceicao *et al.*, 2020)
  1. Maintain BHK-21 cells in 25 ml DMEM-10% in a 75 cm^2^ tissue culture flask. Seed BHK-21 cells in 24-well plates at 1 × 10^5^/well in DMEM-10%. Incubate overnight at 37°C, 5% CO_2_.
  2. Ensure plated cells are at 60-80% confluency to ensure optimal transfection efficiency. Set up transfection mixes in 100 µl of Opti-MEM along with 500ng of different species of ACE2-expressing constructs or an empty vector control (*e.g*., pDISPLAY). Bring the Tran*IT*-X2 transfection reagent to room temperature before use and add 3 µl for every 1 µg of DNA directly to the tube and gently flick the tube to mix. Incubate at room temperature for 20 min.
  3. Add 100 µl of the transfection mix dropwise to each well of the pre-plated BHK-21 cells and incubate overnight at 37°C, 5% CO_2_.
  4. Remove the media containing the transfection mix from the wells and add 0.5 ml of 2 mM EDTA in PBS per well to harvest the transfected cells. Transfer to a bijou and dilute cells to 2 × 10^5^/ml with DMEM-10%.
  5. Seed 100 µl of diluted cells (2 × 10^4^ per well) into a flat, white-bottomed 96-well plate and incubate overnight at 37°C, 5% CO_2_.
  6. Remove media from cells and infect with SARS-CoV-2 or SARS-CoV pseudoparticles equivalent to 10^6^-10^7^ relative light units (RLU), or the no GP control at the same dilution and incubate for 48 h at 37°C, 5% CO_2_.
  7. Remove the media from the wells and add 50 µl of Bright-Glo^TM^ diluted 1:1 with serum free, phenol red free DMEM. Incubate the plate in the dark for 5 min then read on a GloMax Multi+ Detection System under the luminescence protocol with 0.5 s integration.
  8. Export the CSV file generated on a USB flash drive for analysis using Microsoft Excel and plot data on GraphPad Prism.
- Neutralisation assay using SARS-CoV-2 and SARS-CoV pseudotyped virus particles
  1. Prior to setting up neutralization assays, seed HEK293T cells at a density of 7.5 × 10^5^ per well in a 6-well plate in a total of 3 ml of DMEM-10%. Incubate overnight at 37°C, 5% CO_2_.
  2. Ensure plated cells are at 60-80% confluency to ensure optimal transfection efficiency. In a sterile 1.5 ml Eppendorf tube, add 200 µl of Opti-MEM along with 500 ng of human ACE2 plasmid per well to be transfected. Bring the Tran*IT*-X2 transfection reagent to room temperature before use and add 2 µl for every 1 µg of DNA directly to the tube and gently flick the tube to mix. Incubate at room temperature for 20 min.
  3. Add 200 µl of the transfection mix dropwise per well of pre-plated cells and incubate overnight at 37°C, 5% CO_2_.
  4. Set up neutralisation assays by diluting sera/monoclonal antibodies (mAbs)/inhibitors considering the dilution series to be used and the final volume after addition of pseudoparticles. For example, sera to be titrated using a 2-fold dilution series starting at a 1:10 dilution would require 10 µl sera per well in 100 µl serum free DMEM. The same is applicable for mAbs or inhibitors with a known concentration.
  5. Add 100 µl of diluted sera/mAbs/inhibitors in triplicate to the top row of a flat white-bottomed 96-well plate. Add 50 µl of serum free media to all remaining wells. Remove 50 µl from the top row and titrate 2-fold down the plate, mixing well before each titration. Do not titrate into the bottom row. This whole row will be used as the untreated control.
  6. Thaw an aliquot of SARS-CoV or SARS-CoV-2 pseudoparticles and dilute in serum free DMEM, equivalent to ~10^6^ RLU and add 50 µl per well, including the untreated controls. Incubate for 1 h at 37°C, 5% CO_2_.
  7. Remove the media from the 6-well plates transfected with human ACE2. Add 1 ml of DMEM-10% per well and harvest the transfected cells. HEK293T cells have low adherence, so come off the plate easily; therefore, the force of the pipetted liquid should be sufficient to harvest cells (see B4 above). Transfer to a 50 ml Falcon and dilute cells to 2 × 10^5^/ml with DMEM-10%.
  8. Seed 100 µl of diluted cells (2 × 10^4^ per well) onto each well containing sera/mAb/inhibitor with pseudoparticles and the untreated controls. Incubate for 48 h at 37°C, 5% CO_2_.
  9. Remove the media from the wells and add 50 µl of Bright-Glo^TM^ diluted 1:1 with serum free, phenol red free DMEM. Incubate the plate in the dark for 5 min then read on a GloMax Multi+ Detection System under the luminescence protocol with 0.5 s integration.
  10. Export the CSV file generated on a USB flash drive for analysis using Microsoft Excel and plot data on GraphPad Prism.

## Data analysis

- Testing SARS-CoV-2 and SARS-CoV pseudoparticle infectivity
  1. After preparing a batch of pseudoparticles, their infectivity can be tested by titrating them on target cells that have been transfected to express the host receptor (ACE2) of the pseudotyped attachment protein (Spike) for SARS-CoV and SARS-CoV-2. Undiluted pseudotyped virus (“1”) is titrated 10-fold with DMEM-10% down a 96-well plate in triplicate (“10”, “100”, “1,000” *etc*.) (**Figure 1A**).
  2. Measuring the luciferase signal of the pseudoparticles will generate a CSV file that can be exported onto a USB flash drive and analysed on Microsoft Excel. These results can then be plotted on GraphPad Prism to show the mean ± SD for each pseudoparticle.
  3. The no GP negative control serves as an indication of background luciferase signal, and only values above this at each corresponding dilution should be considered as a true luciferase signal for the pseudoparticles being tested (**Figure 1B, black line **). Generally, a minimum of ~2 log dynamic range between the no GP and the pseudotyped virus and a RLU signal between 10^5.5^ and 10^7.5^ RLU (**Figure 1B, shaded area **) is sufficient for use in subsequent assays. The titration series will also help to determine the lowest usable dilution of the pseudoparticles to still obtain meaningful luciferase values above the background. Of note: the following data to be discussed was generated for illustrative purposes only.
  4. For example, when considering the luciferase values obtained for SARS-CoV, although these are above the no GP control at the highest dilution, the difference between the two is only ~1 log, which falls outside our criteria for use (**Figure 1B, blue line **). When titrating the pseudoparticles, the luciferase values also fall quite quickly below the lower limit of the workable range at a 1:10 dilution and to the same values as the no GP control by the 1:1000 dilution, making the titre of this preparation of SARS-CoV pseudoparticles unsuitable for use in subsequent assays (**Figure 1B**).
  5. In comparison, the luciferase signals obtained for SARS-CoV-2 are ~2 log above the background no GP control, and a dilution of 1:10 of the pseudoparticles would be within the workable range for use in subsequent assays, which is lost at a 1:100 dilution (**Figure 1B, orange line **).
  6. The VSV-G pseudoparticles are a positive control within the assay, where the luciferase values observed should be above 10^7^ RLU (**Figure 1B, green line **).
    **Figure 1.**
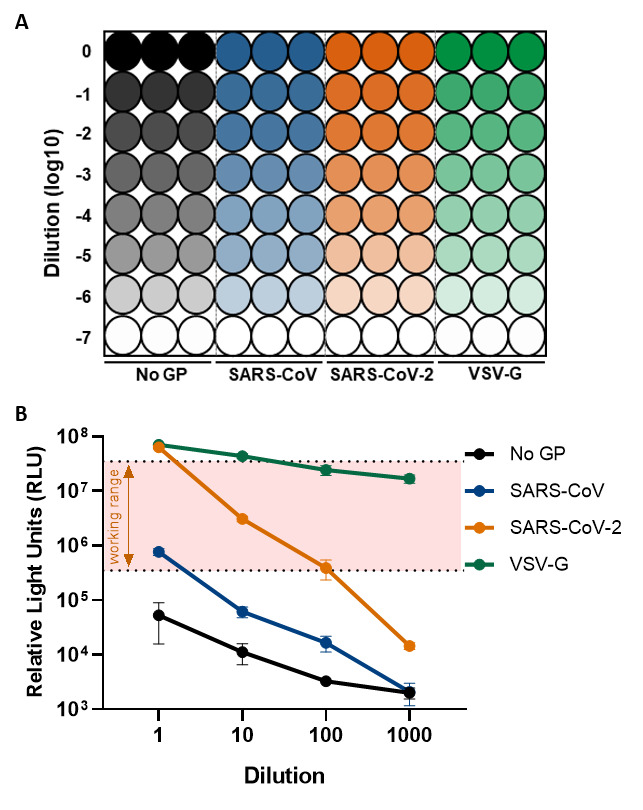
Testing the infectivity of SARS-CoV and SARS-CoV-2 pseudoparticles. (A) For SARS-CoV and SARS-CoV-2 pseudoparticle titrations, 10-fold serial dilutions of the supernatant are used to infect HEK293T cells transiently expressing the human ACE2 receptor in a white flat-bottomed 96 well-plate. Negative (no GP) and positive (VSV-G) controls are also included in the experiment. Each condition is tested in triplicate. **(B)** Two days after infection, signal luciferase values are measured and plotted as the mean ± SD. The no GP control is indicative of the background, and only pseudoparticle values above this should be considered as true infectivity, matched at each dilution of the virus. The use of pseudoparticles for subsequent neutralisation assays and receptor usage screens should show ~2 log dynamic range between the no GP and pseudotyped virus and fall between a working range of 10^5.5^ to 10^7.5 ^(shaded area).
- ACE2 receptor usage screen using SARS-CoV-2 and SARS-CoV pseudotyped virus particles
  1. Infection of cells expressing various species ACE2 receptors with SARS-CoV or SARS-CoV-2 pseudoparticles set up in triplicate yields luciferase signals that can be plotted alongside each other (mean ± SD) to depict raw values. These data also give an idea of the general trend of receptor tropism across different viruses (**Figure 2A**).
  2. For example, water buffalo and goat ACE2 permit the entry of SARS-CoV and SARS-CoV-2 pseudoparticles well, which is less evident for little brown bat ACE2. Differences between viruses can be observed for civet ACE2, which permits the entry of SARS-CoV more efficiently than SARS-CoV-2 (**Figure 2A**). These experiments should be conducted at least three times on three separate occasions, with representative data shown. A subset of ACE2 receptors are shown in **Figure 2A**, but a more in-depth, wider analysis can be found in Supplementary Figure 3A and 3C inConceicao *et al.* (2020).
  3. Two negative controls are set up in this screen. The first is an empty vector control (pDISPLAY) to ensure any signal measured is solely from overexpression of the ACE2 receptor. The second is infection of cells with the no GP control pseudoparticle preparation to ensure luciferase signals can be attributed to the pseudotyped viruses and provides a baseline for the background (**Figure 2A**).
  4. The raw luciferase signals can then be used to determine the relative usage of non-cognate host ACE2 receptors (water buffalo, civet, goat, little brown bat) to a known or cognate host receptor, in this case, human ACE2. The mean percentage from three separate experiments performed on different days are used to obtain these values. The luciferase value for human ACE2 is set to 100%, and the luciferase values for unknown host receptors and the negative control are then expressed as a percentage relative to human ACE2.
  5. These results can also be shown as a heatmap using a colour gradient to show different trends of receptor usage. For example, human ACE2 (100%) is set as green. Expression lower than this is shaded from green to red, indicative of poorer ACE2 usage relative to human ACE2. Values above 100% are shown as a darker green, suggestive of ACE2 usage equivalent to or greater than human ACE2 (**Figure 2B**). A subset of ACE2 receptors are shown in **Figure 2B**, but a more in-depth, wider analysis can be found in Figure 2 A inConceicao *et al.* (2020).
  6. Further analysis can be carried out to compare the receptor tropism of different species ACE2 between SARS-CoV and SARS-CoV-2 by plotting the percentage values for each virus against each other on an *xy* scatter graph, calculating the Pearson’s correlation coefficient, and plotting a linear line of regression fitted with 95% confidence intervals (data not shown). An example of such analysis can also be found in Supplementary Figure 5 inConceicao *et al.* (2020).
    **Figure 2.**
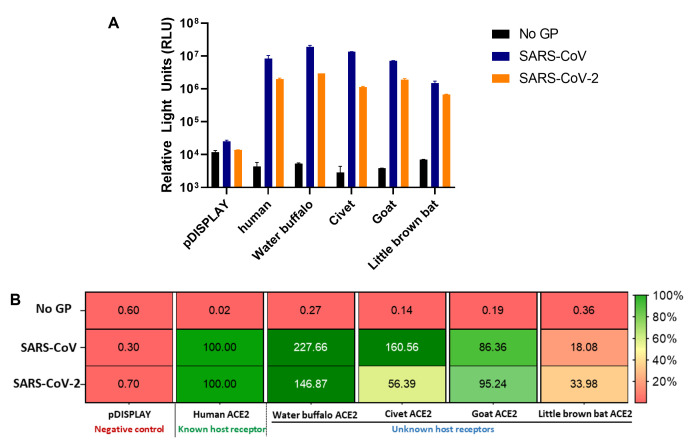
ACE2 receptor usage screen using SARS-CoV and SARS-CoV-2 pseudoparticles. In our study, pseudoparticles were used as a surrogate to live viruses to assess receptor tropism of SARS-CoV and SARS-CoV-2 with different species of ACE2 receptors. Pseudoparticles were employed to assess SARS-CoV and SARS-CoV-2 Spike glycoproteins’ usage of ACE2 receptors from different species and presented as **(A)** raw luciferase signal values or **(B)** a percentage relative to human ACE2. Negative controls were included for pseudoparticles bearing an empty vector control (No GP) or mock-transfected with an empty vector in place of an ACE2 receptor (pDISPLAY). Data are presented as mean ± SD of triplicate values, with each experiment performed three times on three separate occasions, and representative data shown.
- Neutralisation assay using SARS-CoV-2 and SARS-CoV pseudotyped virus particles
  1. Neutralisation assays using pseudotyped viruses are a low-biocontainment alternative to using live virus and can be performed in a relatively high-throughput manner. These neutralisation assays can be performed on mAbs, sera or any other drug or inhibitor that has the potential to inhibit viral entry. The data discussed herein have been generated for illustrative purposes only.
  2. Inhibitors (mAbs/sera/drugs) of a known concentration can be titrated down a 96-well plate in triplicate to determine the extent of inhibition of SARS-CoV-2 entry. This is done by taking an average of the untreated controls and expressing the RLU values for each individual replicate of the mAb of interest relative to this. These can then be plotted as the mean ± SD and should be repeated a minimum of three times, with representative data shown:
    (RLU individual replicate of mAb/RLU average of untreated) × 100
  3. The inhibitory concentration of 50% (IC50) should be indicated on a graph along with the untreated, no mAb control (100%). Values below this IC50 line are indicative of S-mediated inhibition of entry, which can be calculated at each concentration. For example, mAb2 is able to inhibit SARS-CoV-2 S entry by ~80% (20% of untreated) at 100 µg/ ml. The values obtained for mAb2 at all concentrations tested are below the IC50 value, so lower concentrations would need to be tested to determine the limit of inhibition. In contrast, mAb3 inhibits SARS-CoV-2 S entry by ~90% at 100 µg/ml, but at 12.5 µg/ml, the inhibition is now above the IC50 threshold (**Figure 3A**).
  4. There may also be examples of mAbs that do not inhibit SARS-CoV-2 S, as with mAb1. It may be possible that when inhibition of entry is not observed, a slight increase above the 100% threshold is seen. The mechanisms causing this increase are still unknown and under investigation, but for the purposes of this assay, the conclusion that the mAb does not neutralise SARS-CoV-2 S is sufficient (**Figure 3A**). Examples of this sort of analysis can be found inThakur *et al.* (2021).
  5. When determining the inhibition of viral entry from individuals who have antibodies against SARS-CoV-2 S, whether that be following natural infection or vaccination, a neutralising antibody titre is usually calculated to enumerate the level of SARS-CoV-2 S neutralisation. The simplest method of calculating this is by calculating the average RLU of the untreated controls and determining the IC50 value, *i.e*., 50% of the no sera control. The neutralisation titre is then calculated as the inverse of the dilution at which there is 50% inhibition of the no sera luciferase values in all triplicate wells. These titres can then be tabulated or plotted on a log scale.
  6. For example, the titre of 256 for serum sample 1 and 512 for serum sample 3 indicates that IC50 was calculated at a dilution of 1:256 and 1:512, respectively. The conclusion that could be drawn from this is that serum sample 3 is able to neutralise SARS-CoV-2 S-mediated entry more efficiently than serum sample 1 and therefore has higher antibody titres (**Figure 3B**).
  7. For serum sample 4, this value has been plotted as 1,024, which is the upper limit of detection (ULoD) for this assay. This means that this sample was able to inhibit 50% of the luciferase signal in all wells and at the lowest dilution that was tested. This serum would have to be retitrated with a broader dilution series to determine the neutralisation titre. For serum sample 2, none of the wells in the dilution range yielded a recordable IC50. The neutralisation titre is therefore plotted as an arbitrary value below the lower limit of detection (LLoD), which in this case is 40, but would be reported as <40 as the true titre is unknown (**Figure 3B**). Examples of IC50 neutralisation titres using this method can be found in Figure 4F inThakur *et al.* (2021) and in Figure 2D and 2E inGraham *et al.* (2020).
  **Figure 3.**
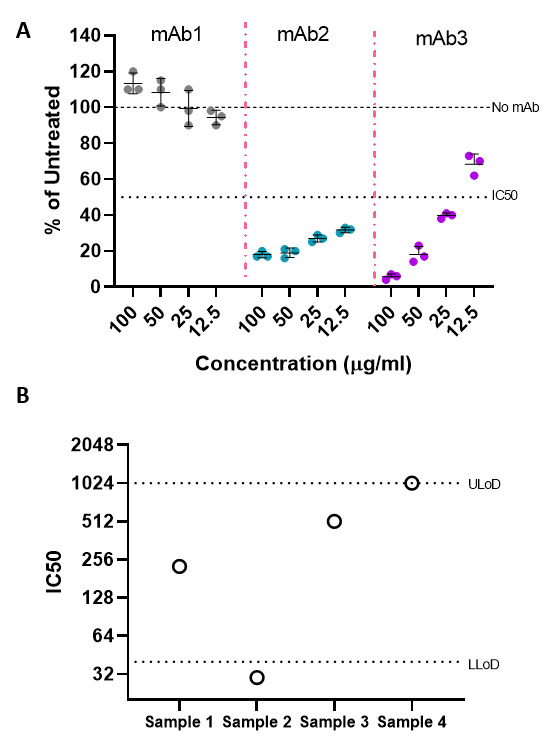
Neutralisation assays using SARS-CoV-2 pseudoparticles. SARS-CoV-2 Spike neutralisation assays were performed in the presence of **(A)** monoclonal antibodies (mAbs), presented as a percentage relative to untreated controls or **(B)** sera samples, with data expressed as neutralising titres. SARS-CoV-2 pseudoparticles were incubated with mAbs or sera for 1 h prior to addition of human ACE2-expressing HEK293T cells. Inhibition of SARS-CoV-2 Spike-mediated viral entry was determined by calculating the concentration (mAbs) or dilution (sera) at which there is a 50% reduction in luciferase signal (IC50). Data represent the mean ± SD of triplicate values, with each experiment performed three times on three separate occasions, and representative data shown.

## Notes

1. Procedure A, step 11 mentions centrifugation of pseudoparticle preparations prior to use in subsequent assays to remove cellular debris. Other protocols require further filtration of pseudoparticles using a 0.45 µm filter before storage. This step is not carried out in our lab, as we have observed a reduction in infectivity following filtration.
2. Manufacturers usually recommend an optimal confluency of 60-80% for transfection. Therefore, it may be necessary to change the seeding density depending on the characteristics of the cells used. For example, if the cell types used are larger (*e.g*., BHK-21 cells are larger than HEK293T cells) or have a high doubling rate, we recommend starting at a lower seeding density. On the other hand, if the cells are smaller, or have a slower growth rate, and are difficult to reach confluency (*e.g*., Calu3 cells) or indeed are suspension cells, you may want to start with a higher seeding density. In both instances, we recommend testing different seeding densities to find the optimal for any given experiment.
3. An example of optimal confluency of HEK293T cells prior to infection (60-80%) is shown in **Figure 4**. Cells should be evenly distributed across the well (*i.e*., no clumping or aggregation in one area), with visible gaps in the monolayer (**Figure 4A**). The cell morphology of the HEK293T cells should appear flat and polygonal at confluence, which indicates adherence to the plastic (**Figure 4B**).
  **Figure 4.**
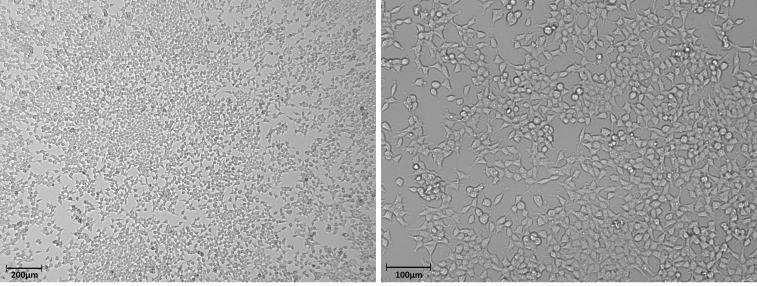
Brightfield image to illustrate 60-80% optimal confluency of HEK293T cells. HEK293T cells were seeded at 7.5 × 10^5 ^in a 6-well dish in 3ml of DMEM-10%. On the following day, cells should be between 60-80% confluent for optimal transfection. **(A)** Example of ~80% confluent HEK293T at 4× magnification, with cells appearing evenly distributed across the well and visible gaps. **(B)** Higher power magnification (10×) of HEK293T cells where cells should appear bright, flat and polygonal prior to transfection.
4. The concentrations of DNA used for transfection at various steps have been optimised for use in these assays. It is important that any plasmids used are optimised to account for variability in vector platform and codon-optimisation.
5. A higher signal (in the 10^4^-10^5^ RLU range) in the no GP control can sometimes be seen, likely the result of non-specific uptake of ‘bald’ pseudoparticles or debris from producer cells. To reduce this background signal, care should be taken to ensure producer cells are not transfected at low confluence as this can cause cytopathic effects (CPE) to develop.
6. All the experiments conducted here were performed with over-expressed ACE2 only. Co-expression of the serine protease TMPRSS2, which is required for S2 protein cleavage to S2’, can facilitate the fusion of viral and cellular membranes and cleavage of the Spike protein (Hoffmann *et al.*, 2020). TMPRSS2 was not included in our host range assays as we wanted to specifically examine the effects of different ACE2s – indeed, over-expression of TMPRSS2 led to ACE2 restrictions being masked (Conceicao *et al.*, 2020).
7. Other formulae can be used to determine the IC50 value, yielding different titres. This is acceptable if the same method is used throughout analyses and the method used are described in full. Other formulae for calculating neutralisation titres include (1) using a non-linear regression analysis tool on GraphPad Prism after plotting data on an *XY* graph to interpolate neutralisation values (Ferrara and Temperton, 2018), (2) interpolating the point at which infectivity is reduced to 50% of the value of a no serum control sample using a fixed formula (Logan *et al.*, 2016), and (3) determining the highest dilution at which complete neutralisation is seen in all replicate wells and considering other wells that also show neutralisation. Neutralisation is then calculated by inputting these values into a Spearman Karber formula (Lambe *et al.*, 2021).
8. Neutralisation titres do not always need to be recorded as IC50 values. Other cut-off points can be chosen dependent on the level of neutralisation expected in a given assay, and to provide a more stringent measure of neutralisation (*e.g*., 80% neutralisation, IC80).
9. The surface expression of different ACE2 receptors may differ, which may affect the level of Spike-ACE2 interaction leading to misinterpretation of results. Therefore, it is important to investigate and normalise the cell surface expression of the ACE2 receptors used. The mammalian ACE2 receptors described and used herein were HA-tagged at the C-terminus, which allowed detection of surface expression by flow cytometry. Additionally, protein expression was assessed by Western blotting (Conceicao *et al.*, 2020).

## Recipes

1. DMEM-10%
DMEM supplemented with 10% FBS, 1% penicillin/streptomycin 10,000 U/ml, and 1% 100 mM sodium pyruvate, cultured at 37°C with 5% CO_2_.
2. Working solution of 1 mg/ml PEI
  - Weigh the viscous liquid to get 50 mg/ml in water (*e.g*., 0.42 g PEI + 8.4 ml water) and transfer to a sterile 50 ml Falcon.
  - Place Falcon in a water bath set to 50°C and gently pipette up and down using a 1 ml pipette until fully dissolved.
  - Dilute to 1 mg/ml with water (*e.g.*, take 0.5 ml of your 50 mg/ml stock and add 24.5 ml water)
  - The solution in its current state will be very basic. Adjust pH to 7 using diluted hydrochloric acid.
  - Filter through a 0.22 µm filter and aliquot into 1.5 ml Eppendorf tubes.
  - Store at -20°C long-term and at 4°C for up to one month while in use.
